# In the Early Stages of Diabetes, Rat Retinal Mitochondria Undergo Mild Uncoupling due to UCP2 Activity

**DOI:** 10.1371/journal.pone.0122727

**Published:** 2015-05-07

**Authors:** Ixchel Osorio-Paz, Salvador Uribe-Carvajal, Rocío Salceda

**Affiliations:** 1 Departamento de Neurodesarrollo y Fisiología, Instituto de Fisiología Celular, Universidad Nacional Autónoma de México, México Distrito Federal, México; 2 Departamento de Genética Molecular, Instituto de Fisiología Celular, Universidad Nacional Autónoma de México, México Distrito Federal, México

## Abstract

In order to maintain high transmembrane ionic gradients, retinal tissues require a large amount of energy probably provided by a high rate of both, glycolysis and oxidative phosphorylation. However, little information exists on retinal mitochondrial efficiency. We analyzed the retinal mitochondrial activity in *ex vivo* retinas and in isolated mitochondria from normal rat retina and from short-term streptozotocin-diabetic rats. In normal *ex vivo* retinas, increasing glucose concentrations from 5.6mM to 30mM caused a four-fold increase in glucose accumulation and CO_2_ production. Retina from diabetic rats accumulated similar amounts of glucose. However, CO_2_ production was not as high. Isolated mitochondria from normal rat retina exhibited a resting rate of oxygen consumption of 14.6 ± 1.1 natgO (min.mg prot)^-1^ and a respiratory control of 4.0. Mitochondria from 7, 20 and 45 days diabetic rats increased the resting rate of oxygen consumption and the activity of the electron transport complexes; under these conditions the mitochondrial transmembrane potential decreased. In spite of this, the ATP synthesis was not modified. GDP, an UCP2 inhibitor, increased mitochondrial membrane potential and superoxide production in controls and at 45 days of diabetes. The role of UCP2 is discussed. The results suggest that at the early stage of diabetes we studied, retinal mitochondria undergo adaptations leading to maintain energetic requirements and prevent oxidative stress.

## Introduction

The vertebrate retina possesses the highest respiratory rate of any other tissue examined in vitro [1]; this organ requires a large amount of energy, which is primarily used to maintain the ionic gradients across cell membranes. Retina may suffer irreversible damage from large variations in oxygen or glucose concentrations [2]. Mitochondria are the main source of energy in the cell and are considered to be a major source of reactive oxygen species (ROS), the formation of which is unavoidable during oxidative metabolism [3]. In spite of this, relatively few studies on mitochondrial function in retina have been undertaken [4]. In diabetic retinopathy, mitochondrial activity has been postulated to increase, leading to oxidative stress [5,6]. In rats, changes in mitochondrial activity and DNA damage have been observed after long-time diabetes [3,7]. The mechanism leading to these changes has not been identified. Therefore, to get insight into the role of mitochondria in retinal physiopathology, we isolated mitochondria from normal rat retina and compared their activities with mitochondria from rats in the early stages after diabetes induction.

## Material and Methods

### Animals

Adult Long Evans rats were used in this study. Diabetes was induced with a single intraperitoneal injection of streptozotocin (90mg/Kg), freshly dissolved in citrate buffer pH 4.5. Animals were not treated with insulin; they were maintained at 21°C on a 12h alternating light-dark cycle and allowed food and water *ad libitum*. Animals were considered diabetic when values were equal or higher than 300mg/dl the day after stretptozotocin administration. At the moment of sacrifice blood glucose levels varied from 300–600 mg/dl in all groups studied. Normal rats had blood glucose levels of 90–120 mg/dl. Diabetic and control matched animals were sacrificed by decapitation after 7, 20 and 45 days of STZ administration. The enucleated eyes were hemisected and the anterior part was removed; the retinas were gently peeled away using fine forceps. The retinas for one rat were used for each determination, except for mitochondria preparation, in which the retinas from 3–5 rats were used in each experiment.

All procedures were conducted in accordance with the Mexican Institutes of Health Research (DOF. NOM-062-Z00-1999) and the National Institutes of Health Guide for the Care and Use of Laboratory Animals (NIH publication No. 80–23, revised 1996), as well as the Research Association in Vision and Ophthalmology Statement on the Use of Animals in Ophthalmic and Vision Research. The experimental protocol was approved by the Committee on the Ethics in Animal Experimentation at our Institution. All efforts were made to minimize animal suffering, and to reduce the number of rats used.

### CO_2_ production from glucose

The rates of ^14^CO_2_ production from ^14^C-glucose by retina were determined as described previously [8,9]. Retinas were incubated at 37°C in gassed (95%O_2_-5%CO_2_) Ringer buffer (NaCl, 118mM; KCl, 4.7mM; KH_2_PO_4_, 12mM; MgSO_4_, 1.17mM; NaHCO_3_, 25mM; glucose, 5.6mM or 30mM, pH 7.4), containing 0.25–0.5μCi of [1-^14^C] glucose (58mCi/mmol) or [U-^14^C] glucose (265mCi/mmol), (Amersham, UK). The metabolic activity was stopped by the addition of H_2_SO_4_. Released ^14^CO_2_ was trapped in center well containing benzethonium hydroxide and its radioactivity was determined in a liquid scintillation spectrometer. Relative specific activity of ^14^CO_2_ was considered as the specific activity of ^14^C-glucose in the medium. Accumulated glucose was determined by the radioactivity present in the tissue.

### Adenosine triphosphate content

For ATP content, retinas were rapidly homogenized in cold 6% (w/v) perchloric acid and protein was removed by centrifugation. ATP was determined by an enzyme-coupled reaction to hexokinase (HK) and glucose 6 phosphate dehydrogenase (G6PDH) activities according to Trautschold *et al*. (1988) [10]. The NADPH decrease was followed at 340nm and the concentration was calculated using ε = 6.3 mM^-1^ cm^-1^.

### Mitochondria isolation

Mitochondria were isolated by a procedure described before [11], with some modifications. Six to ten retinas were homogenized in 1ml of the isolation medium (IM): 2mM, Hepes pH 7.4; 220mM, mannitol; 10mM, sucrose; 10mM, taurine; 1mM EDTA, and 1% BSA fat-free fraction V. The homogenate was centrifuged at 650g for 10 min, to improve the mitochondrial content, the pellet was rinsed with 1ml of IM and then centrifuged at 650g; joined supernatants were centrifuged at 8200g for 15 min, the pellet (mitochondrial fraction) was gently resuspended in IM. All procedures were carried out at 4°C, and determinations were conducted within 3 hours of mitochondrial isolation.

The integrity of the outer mitochondrial membrane was assessed by measuring cytochrome c oxidase activity in the presence and absence of n-dodecyl β-D-maltoside, using the Cytochrome c Oxidase Assay Kit from SIGMA. We found 42% integrity of the outer mitochondrial membrane, similar to that reported for mitochondria from other tissues [12].

Mitochondrial structure was assessed by electron microscopy. The mitochondrial fraction was fixed in a 3% glutaraldehyde solution dissolved in a cacodylate buffer pH 7.2 for 2 hours at 4°C, post-fixed in 2% (v/v) osmium tetroxide, dehydrated in graded ethyl alcohol and embedded in epoxy resin. Ultrathin sections were obtained with a Sorval MT6000 ultramicrotome, contrasted with uranyl acetate-lead-citrate, and examined with an electron microscope JEOL JEM 12000 EII. Electron microscopy studies revealed a high purity fraction (75–80%) and mitochondrial morphology similar to that observed in histological studies [13]. (S1 Fig). To obtain this preparation 10mM taurine and 1.0% defatted BSA were added to IM. The quality of this preparation allowed us to study mitochondrial activity from normal rat retina and compare to pathological situations.

### Oxygen consumption

Mitochondrial oxygen consumption was measured with a Clark electrode, Warner/Strath Kelvin in a 100μl chamber. The mitochondrial fraction (50μg protein) was incubated at 30°C, in IM plus 2mM inorganic phosphate (Pi), 20mM KCl, 1mM MgCl_2_, and 10mM glutamate/malate, to establish the resting state (state 4). The active state (state 3) was induced by 100μM ADP. 1mM KCN was added to verify the mitochondrial source of oxygen consumption.

### Mitochondrial respiratory complexes activity

Mitochondrial fractions were frozen and thawed to allow substrate uptake and the activity of complexes I, II and III were measured spectrophotometrically as described by Turnbull [14]. Complex I activity was measured by following the absorbance of NADH at 340 nm (ε = 6.3 mM^-1^ cm^-1^). Mitochondria (100μg protein) were incubated in IM in the presence of 13mM NADH, 25mM potassium phosphate, 5mM MgCl_2_, 2mM KCN, BSA (2.5mg/ml), 65μM ubiquinone and antimycin A (2μg/ml). Complex II activity was determined by following the absorbance of 2,6 *dichloroindophenol (DCPIP) at 600nm (ε = 19.1 mM^-1^ cm^-1^). Mitochondria (20μg protein) were* incubated for 10 min in IM containing 20mM sodium succinate. 2μg/ml Rotenone, 2μg/ml Antimycin A, and 2mM KCN were added to inhibit complexes I, III and IV, respectively. The reaction was started with the addition of 65μM ubiquinone and 50μM DCPIP. Complex III activity was measured in mitochondria (0.5mg protein) incubated in IM, containing 15 μM cytochrome C, 2μg/ml rotenone, 0.6mM dodecyl-β-D-maltoside and 35μM ubiquinol. The absorbance of reduced cytochrome C was monitored at 550nm (ε = 19.1 mM^-1^ cm^-1^). To measure complex IV activity, we followed the rate of oxygen consumption [15]: 100μg of fresh mitochondrial protein were incubated in a potassium phosphate buffer assay (25mM) pH 7.2 containing 50μM tetramethyl phenylenediamine (TMPD), 1μg /ml antimycin A, 5mM ascorbate and 1mM KCN.

### Mitochondrial transmembrane potential

The mitochondrial transmembrane potential (∆ψ) was estimated using safranine-O fluorescence at 586nm/495nm (em-ex), using an (DW2C Aminco Ollis) spectrofluorometer [16]. Mitochondria (100μg protein) were incubated at 30°C in IM containing 10μM safranine-O, 2mM Pi, 20mM KCl, 1mM MgCl_2_. Addition of 5μM carbonyl cyanide *m*-chlorophenyl hydrazone (CCCP) was used to dissipate the ∆ψ. Mitochondria from normal and diabetic rat retinas were performed in parallel and maximal fluorescence was normalized.

### Mitochondrial ATP synthesis

For ATP synthesis, mitochondria (20μg protein) were incubated at 30°C in IM with the following additions: 50mM glucose, 0.5U/ml glucose 6 phosphate dehydrogenase, 350μM NADP^+^, 1.8 U/ml hexokinase, 2mM Pi, 20mM KCl, 1mM MgCl_2_ 10mM glutamate/malate; the reaction was started with 100μM ADP, and the mitochondrial ATP synthesis was followed by the NADPH absorbance at 340nm (ε = 6.3 mM^-1^ cm^-1^). A parallel sample was incubated with oligomycin (10 μg/mg protein) to inhibit the activity of ATP synthase and values were subtracted to the total absorbance change.

### Superoxide production

Superoxide was measured by reduction of nitroblue-tetrazolium (NBT), using a standard curve of superoxide production by the xanthine-xanthine oxidase system [17]. Mitochondria (50μg protein) were incubated at 30°C in IM containing 2mM Pi, 20mM KCl, 1mM MgCl_2_, 10mM glutamate/malate, and 200μM NBT. Reduction of NBT was monitored at 550nm.

### COX and UCP2 expression

Western blots were performed as previously described [18]. A retina was transferred into a lysis buffer (1:3 (p/v): RIPA-Tris buffer (2mM EGTA; 316mM NaCl; 20mM Na_2_MoO_4_; 50mM NaF; 20mM Tris-HCl; 100mM PMSF and 100mM EDTA; 0.1% leupeptine and 0.1% aprotinine; 0.2% SDS and 2% Triton-X100) and maintained under constant shaking for 1 h at 4°C. The sample was resolved on a 10% SDS polyacrilamide gel. Proteins were transferred into PVDF Immobilon membranes (Millipore Corp, Billerica, MA). After being blocked with 5% fat-free milk, the membranes were probed with rabbit anti-uncoupling protein 2 (Alpha Diagnostic Int Inc, San Antonio, TX) or anti-cytochrome C oxidase (COX IV)(1:1000, Cell Signaling Technology, Danvers, MA); followed by horseradish-peroxidase-conjugated secondary antibody (1:10000, Amersham Biosciences Piscataway, NJ). Protein loading was normalized to actin using a monoclonal primary antibody (1:25000, Chemicon, Temecula, CA). The signal was detected by enhanced chemioluminiscence using Chemioluminiscent HRP substrate (Millipore Corp, Billerica, MA). Densitometry was performed with an Alpha DigiDoc RT (Alpha Innotech, San Leandro, CA).

### Protein content

Protein content was determined by the Lowry method, using a kit from Bio Rad, with BSA as standard.

### Statistical analysis

Results were analysed with the one way ANOVA test followed by Tuckey`s and T student analysis using the GraphPad Prism 5 program. The values were considered statistically significant if p < 0.05.

## Results

Retinas from normal rats accumulated glucose proportionally to added glucose. As shown in Table 1, increasing glucose concentration from 5.6mM to 30mM caused a four-fold increase in glucose uptake. High levels of glucose (30mM) increased four-fold the levels in CO_2_ production from mitochondria. Additionally, the amount of CO_2_ produced from [1-^14^C] glucose represents 2–4% of accumulated glucose (Table 1). As [1-^14^C] glucose is also decarboxylated by glycolysis, the contribution of the pentose phosphate pathway (PPP) was estimated. This was achieved by subtracting one sixth of the CO_2_ values obtained. Retinas from 20 day-diabetic rats also accumulated glucose proportionally to glucose concentration in the medium. However 20 day-diabetic retinas from diabetic produced 40% less CO_2_ at high glucose (30mM).

**Table 1:** Rates of [^14^C] Glucose in Normal and Diabetic Rat Retina.

CO_2_ Production (Glucose Oxidized)								
Gucose	Glucose Accumulated		[U-^14^C] glucose		[1-^14^C] glucose		PPP	
	Control	Diabetic	Control	Diabetic	Control	Diabetic	Control	Diabetic
5.6	76 ± 7.3	89 ± 16	17 ± 2.5	14 ± 2.	3 ± 0.4	1.9 ± 0.2*	2.8	2.3
30	406 ±67§	322 ±79§	66 ± 8.5§	42 ± 4§ *	5 ± 0.9§	6 ± 1.2§	11	7

Incubation was carried at 37°C for 20 min in the presence of glucose of different concentrations. The activity of PPP is taken as one sixth of CO_2_ produced from [U-^14^C] glucose. The results are expressed in nmol/mg protein. Values are mean ± SEM for at least five experiments.

*p = 0.02 respect to non diabetic;

§ p≤ 0.03 respect 5mM.

**Fig 1:**
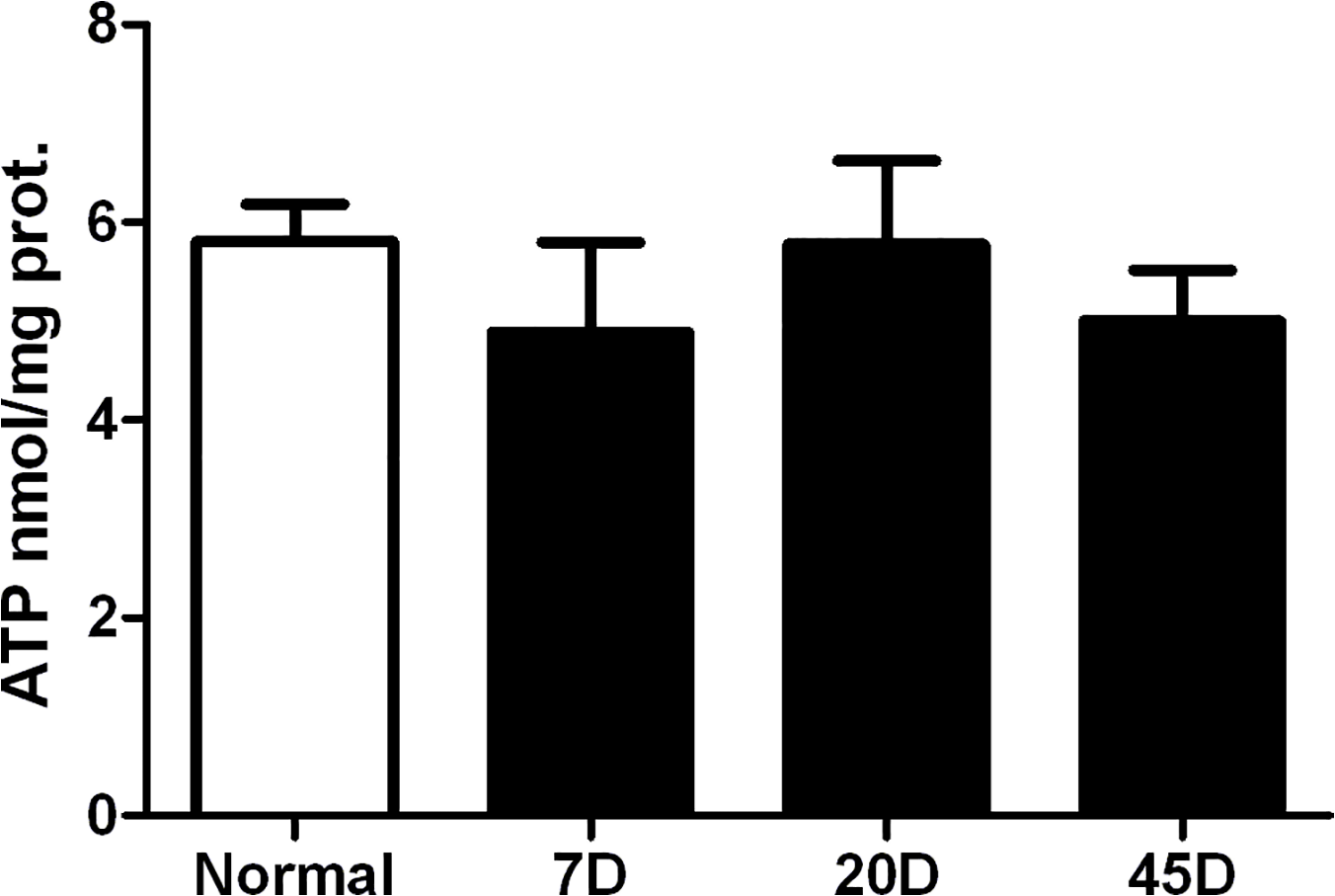
ATP content in rat retina, before treatment and after 7, 20 and 45 days of streptozotocin administration. Values are the mean ± SEM for three to six independent experiments.

The decrease in CO_2_ production by diabetic rats suggested that oxidative phosphorylation was less efficient. Therefore, we analyzed the ATP content of normal and diabetic rat retinas. We found ATP levels of 5.8 ± 0.37 nmol/mg protein in normal retina, values which were similar to those reported previously [19]. In diabetic rat retinas the ATP content was not significantly different to the controls (Fig 1).

### Mitochondrial activity

**Fig 2:**
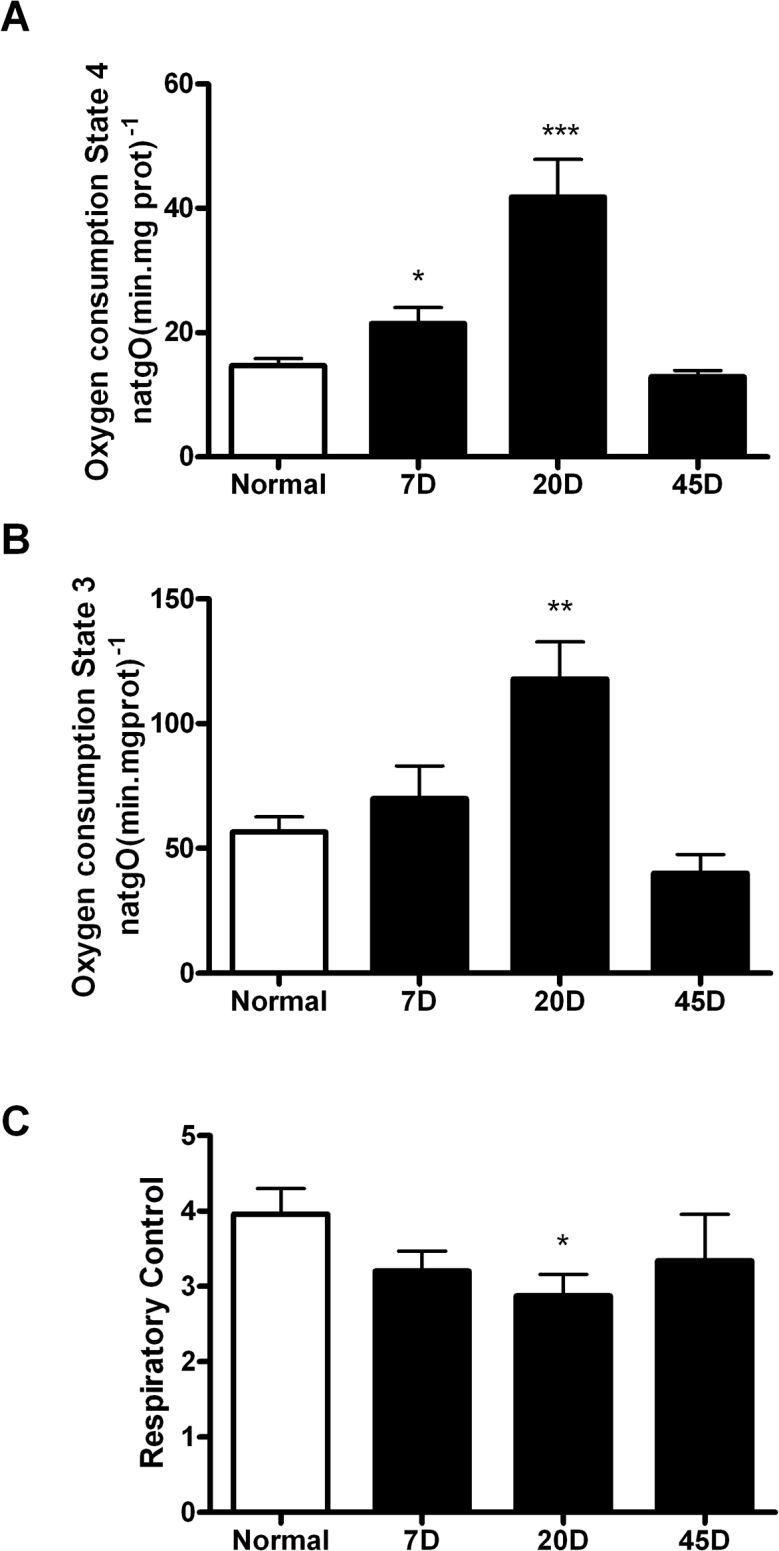
Mitochondrial oxygen consumption. (A) Oxygen consumption was assessed in isolated mitochondria incubated at 30°C in IM with 10mM glutamate /malate as oxidative substrate in state 4 and (B) in active state (3) (100μM ADP). (C) Respiratory control ratio (RC). Values correspond to natgO/min/mg prot ± SEM of at least six independent experiments. p<0.05; ** p<0.005; *** p<0.0005, respect to the normal.

The decreased CO_2_ release led us to evaluate oxidative phosphorylation in isolated mitochondria. In the presence of glutamate/malate, the resting-state (state 4) rate of oxygen consumption in mitochondria from normal rat retinas was relatively high, 14.6 ± 1.1 natgO(min.mg prot)^-1^, and respiratory control (RC) = 4 (S2 Fig). In mitochondria from 7 day-diabetic rats the rate of oxygen consumption in both state 4 and state 3 increased mildly, while RC did not decrease significantly. In contrast, in mitochondria from 20 day-diabetic rats, state 4 increased further, leading to a significant decrease in RC (Fig 2). Interestingly, mitochondria from 45 days diabetic rat retinas exhibited a recovery in the state 4 and state 3 rates of oxygen consumption with the resulting recovery in RC, such that it was similar to control mitochondria.

**Fig 3:**
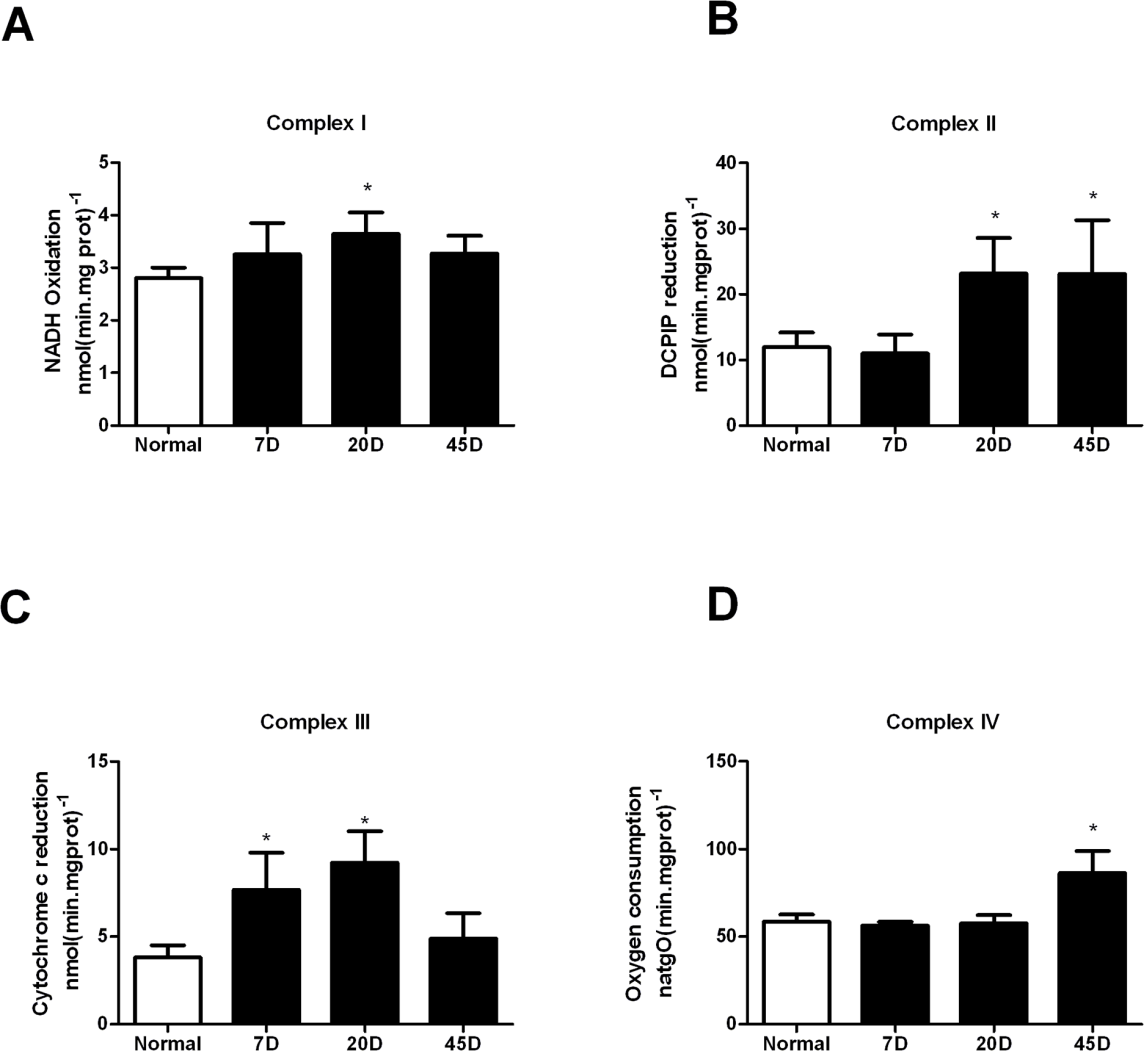
Activity of the respiratory complexes. Complexes activities in isolated mitochondria from normal and diabetic rat retina, were determined as described in Methods. Results are the mean ± SEM of at least four independent experiments. *p<0.05.

Subsequently, we determined the activity of the electron transport respiratory complexes. The different mitochondrial respiratory complexes from normal rat retina activities were as follows: complex I, 2.80 nmol(min.mg prot)^-1^; complex II, 11.95 nmol(min.mg prot)^-1^; complex III 3.8 nmol(min.mg prot)^-1^; and complex IV 58.6 natgO(min.mg prot)^-1^. In the 7 day-diabetic samples, only complex III increased its activity. At 20 day-diabetic, mitochondria exhibited an increase in complexes I, II and III. Then at 45 day-diabetic rats complexes I and III activity returned to normal while complex II activity remained high and only at this stage complex IV increased it activity, to 86.3 natgO(min.mg prot)^-1^ (Fig 3).

**Fig 4:**
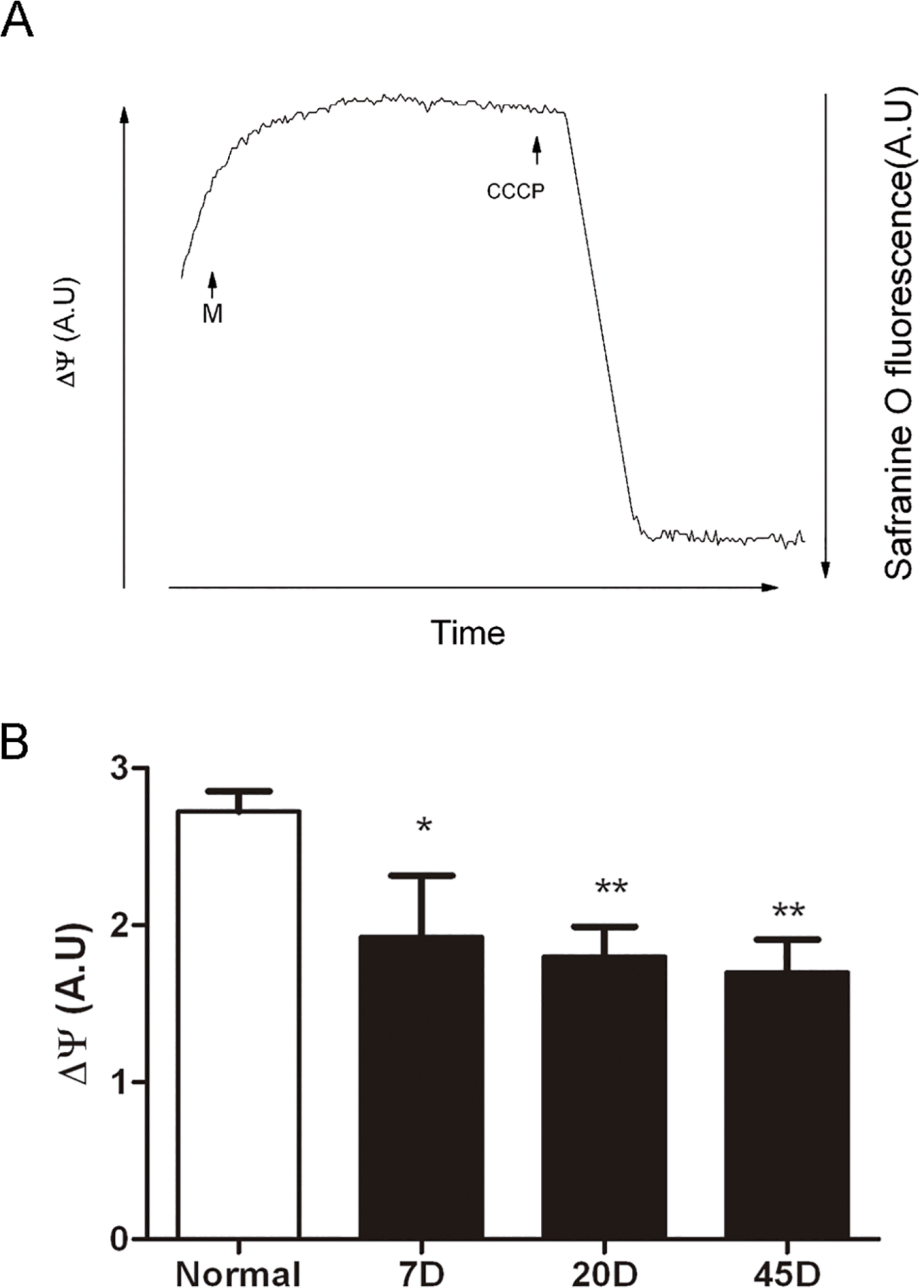
Mitochondrial membrane potential. (A) Representative mitochondrial transmembrane potential trace, (M) mitochondria (100μg protein) from normal rat retina were incubated at 30°C, in IM, monitoring the Safranine O fluorescence. The ∆ψ was dissipated by the addition of 5μM CCCP, as described in Methods. (B) ∆ψ from normal and diabetic (7, 20, 45 days) rat retinas. Values are the mean ± SEM from at least four separated experiments.* p< 0.5, ** p< 0.05.

**Fig 5:**
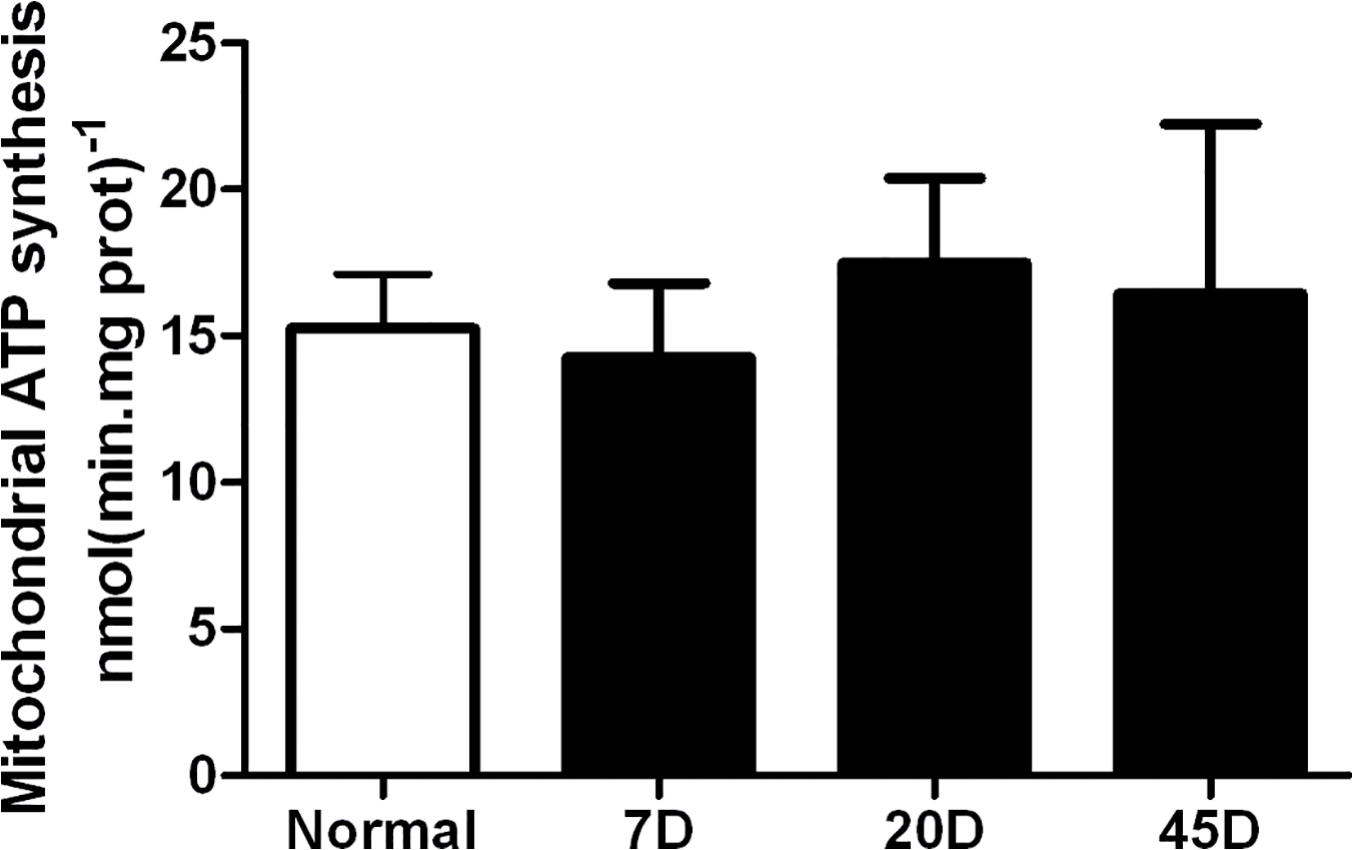
Mitochondrial ATP synthesis. Mitochondrial fraction was incubated at 30°C in IM, using glutamate/malate (10mM) as oxidative substrate and 100μM ADP. The ATP production was determined by NADPH reduction by a coupled reaction with HK and G6P-DH enzymes as described in Methods. Incubation was carried out in the absence and presence of oligomycine (10μg/mg prot.) to obtain the specific ATP produced by ATP synthase. Each value is the mean ± SEM of three to five independent experiments.

In spite of the increase in activity of the mitochondrial complexes, it was observed that ∆ψ was reduced by 30% to 40% in retinas from all 7, 20 and 45 day-diabetic samples (Fig 4). The result suggested that there might be an uncoupling effect produced by hyperglycaemic conditions. To test this, we decided to measure the rate of synthesis of ATP in mitochondria from each group. Unexpectedly, the rate of ATP synthesis, 15.3±2 nmol (min.mg prot)^-1^ was similar in mitochondria from all samples, including normal and diabetic rat retinas (Fig 5).

**Fig 6:**
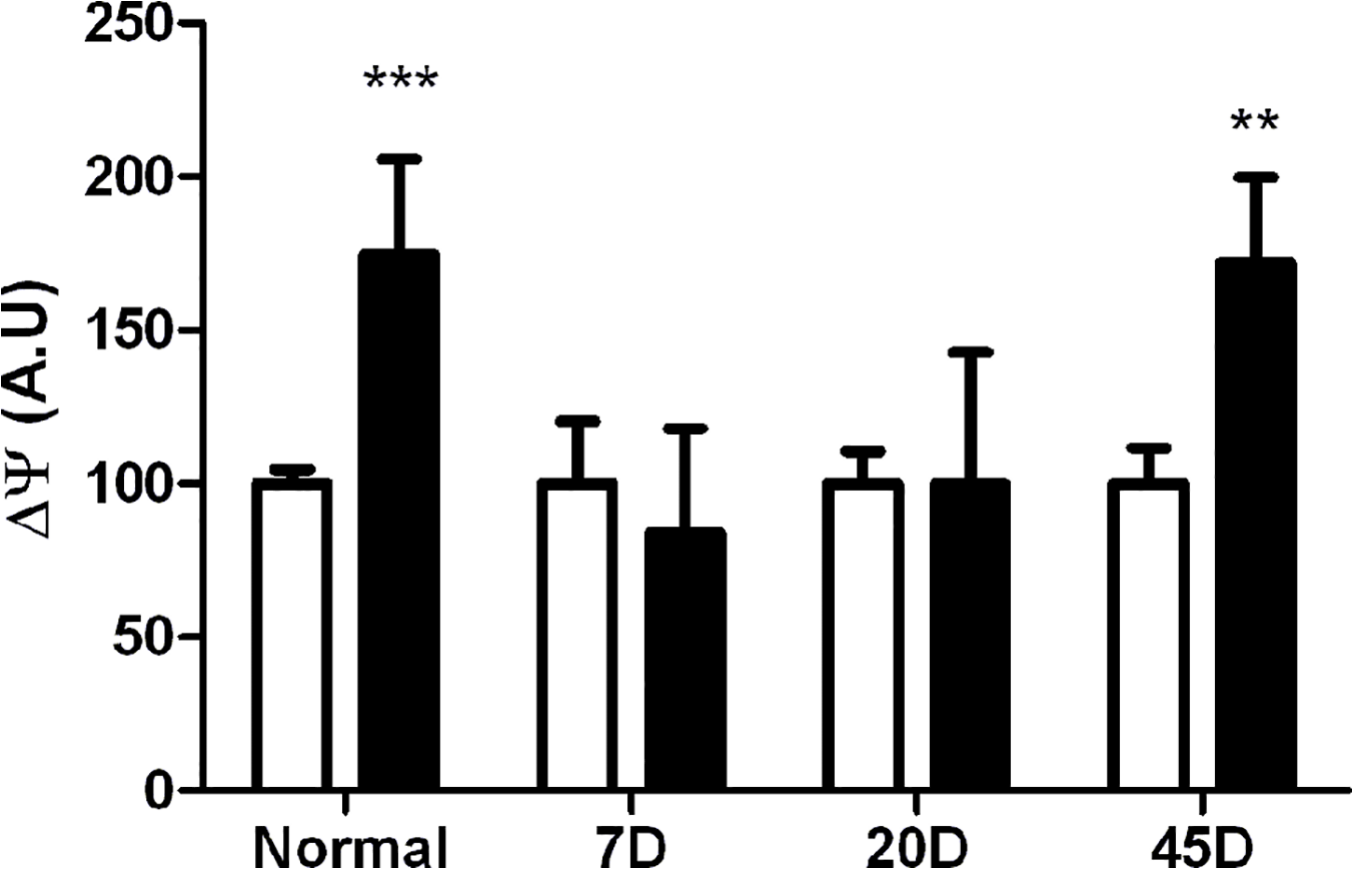
Effect of GDP on mitochondrial transmembrane potential. ∆ψ was determined in the absence (white bars) and presence of 1mM GDP (black bars). Each value corresponds to the average ± SEM of at least three independent experiments.

Diabetes led to different adaptations in mitochondria, such as the different activities of the respiratory complexes and a small, but consistent decrease in ∆ψ. However, these changes do not seem to reflect damage, as the synthesis of ATP did not change. Thus, we decided to seek for an adaptive response in diabetic mitochondria such as the expression of UCP2. In this regard, it has been reported that, in other tissues UCP2 is expressed in order to prevent ROS overproduction [20]. Indeed, UCP2 expression has been reported to decrease the ∆ψ without inhibiting the synthesis of ATP [21]. The activity of UCP2 was tested evaluating the sensitivity of the ∆ψ to GDP an UCP2-inhibitor. In control mitochondria, GDP increased the ∆ψ by 50%, but it lost its effects in the 7-day and 20-day-diabetic mitochondria (Fig 6). Then, at 45 day-diabetic treatment, mitochondria fully recovered the sensitivity to GDP, indicating that 45 day-diabetic mitochondria fully recovered their UCP2 activity.

**Fig 7:**
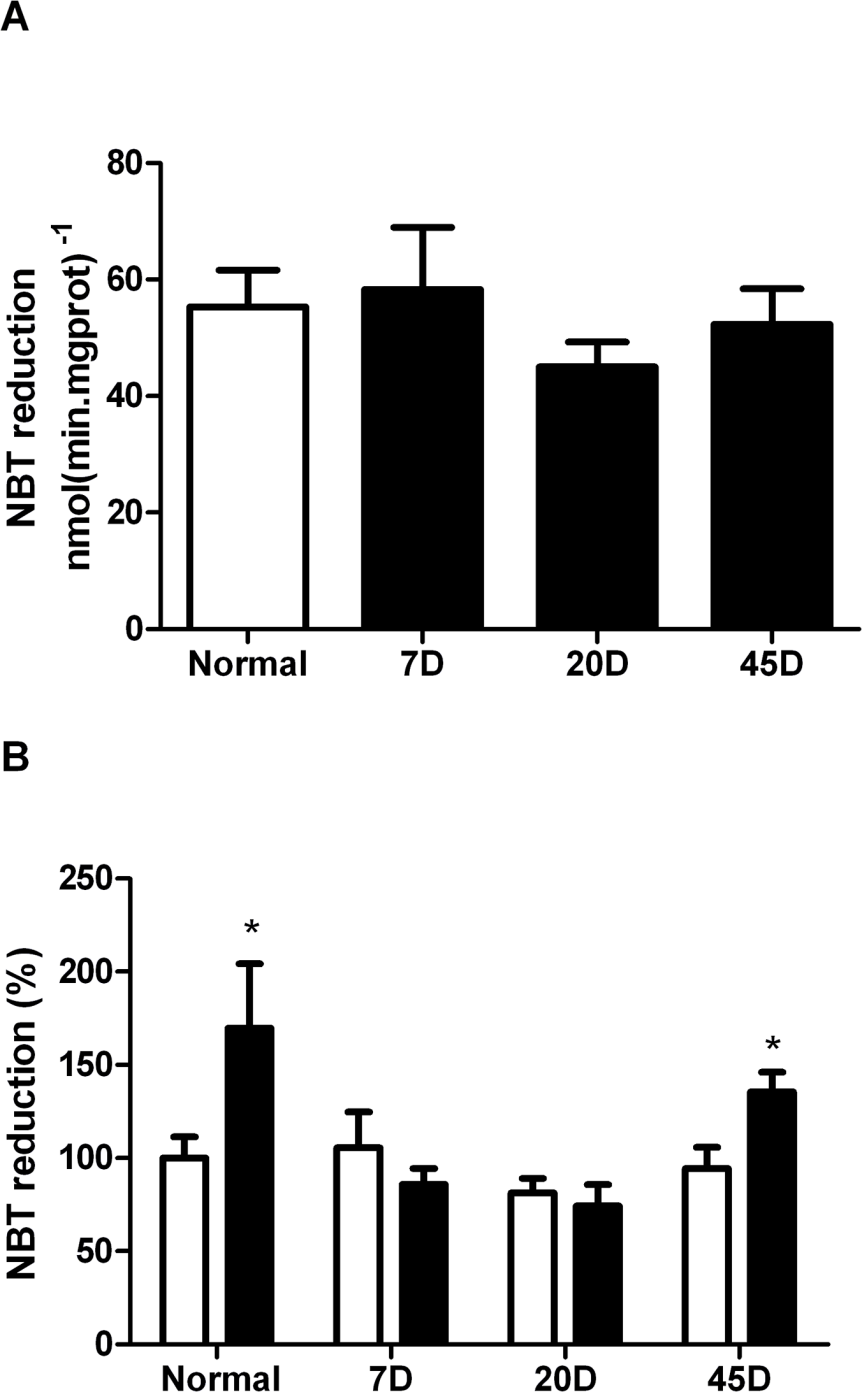
Superoxide production was determined by the reduction of NBT by incubation of isolated mitochondria at 30° in IM at state 4 conditions. (A) Superoxide production from normal and diabetic rat retina. (B) Mitochondria were incubated in the absence (white columns) or in the presence of 1mM GDP (black columns). Each value represents the average ± SEM of three to six independent experiments * p< 0.5.

**Fig 8:**
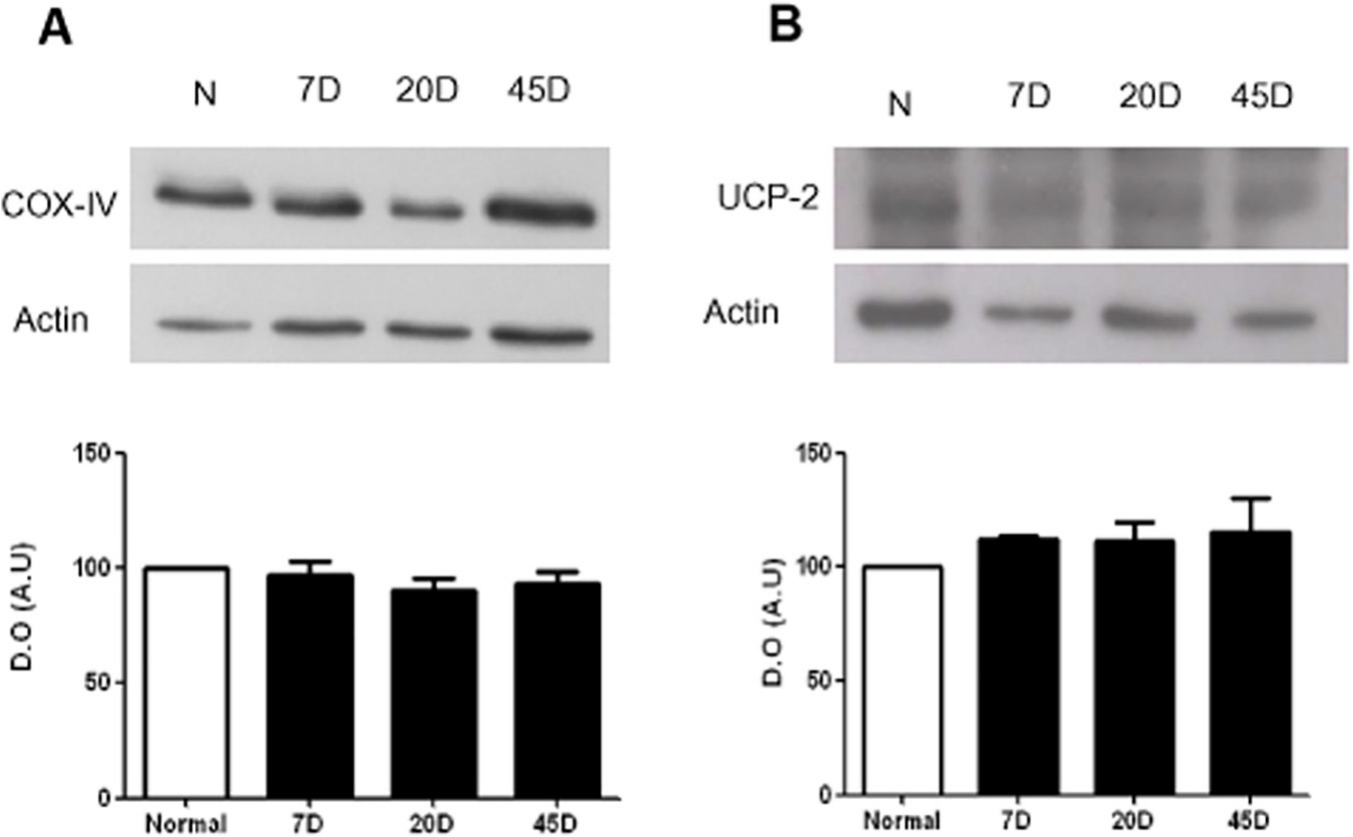
Uncoupling UCP2 and COX expression levels in retina. Western blot of (A) COX-IV and (B) UCP2 in normal, 7, 20 and 45 days after diabetes induction. Upper part, a representative western blot experiment; lower part, densitometric analysis of three to five independent experiments.

To test whether the UCP2 activity correlated with a decrease in ROS production, it was decided to measure superoxide mitochondrial production in each sample in the presence and in the absence of GDP. When we measured the reduction of NBT to yield formazan it was observed that superoxide production in the 7 day and 20 day-diabetic samples was insensitive to GDP. In contrast, in the control and in the 45-day-diabetic sample, GDP induced an increase in ROS production, suggesting that the control UCP2 activity was recovered in the 45 day sample (Fig 7). This recovery in activity did not seem to reflect changes in the expression of the protein, as neither the expression levels of UCP2 nor COX change under any of the conditions tested (Fig 8). Activation of UCP2 and COX in 45-day diabetic mitochondria seems to be a response to the diabetes process that leads to more efficient handling of ROS.

## Discussion

Retinal tissue is outstanding because it requires a large amount of energy and exhibits a high rate of respiration, thus it is particularly vulnerable to abnormalities in energy metabolism [22,23]. Hyperglycemia is a determinant factor in the development of diabetic retinopathy [6], which has been related to a high mitochondrial activity leading in high ROS production and oxidative stress [5,24]. Few studies have focused on the mitochondrial bioenergetics of retina, mainly due to the limiting amount of tissue. This inconvenience was eliminated using microliter assay systems. One of our goals was to isolate enriched retinal mitochondria that allowed us to analyze mitochondrial function: respiratory control, mitochondrial complex activity, ATP synthesis and ∆ψ.

In order to get insight into the normal mitochondrial function and its possible relation with diabetic retinopathy we first evaluated mitochondrial activity in the entire retina by means of glucose oxidation. Our results indicated that all samples of rat retina accumulated glucose proportionally to its extracellular concentration. However, in normal retina, CO_2_ production from glucose oxidation was proportional to its accumulation, whereas in diabetic rat retina the CO_2_ yield was lower. On the other hand CO_2_ production by the PPP pathway did not change [19]. The decrease in CO_2_ production from glucose mediated by diabetic rat retina, suggests a decrease in mitochondrial glucose oxidation, which was not supported by the normal ATP levels observed in diabetic retina. Then, the normal ATP content found in diabetic retina, most likely represent its high glycolytic activity [1]. In fact, we previously reported a high lactate and glycogen content in diabetic rat retina [25,26]. In this respect, retinal endothelial cells incubated in high glucose concentrations, decrease oxygen consumption and increase extracellular acidification [27]. However, utilization of glucose may vary among different type of cells, since retinal pericytes incubated in high glucose concentrations, decrease both oxygen consumption and acidification [28].

Interesting, recent studies showed that UCP2 activity decreases mitochondrial glucose oxidation, favoring aerobic glycolysis and promotes oxidation of fatty acids and glutamine by exporting C4 metabolites from mitochondria to the cytosol [29–31]. In fact, in spite of the lower rate of glucose oxidation in the diabetic rat retina, we observed increased activity of all mitochondrial respiratory complexes plus a more active respiratory chain. The higher state IV respiration rate resulted in a RC below normal at 20 days of diabetes. The increase in the rate of oxygen consumption and the activities of each respiratory complex was not due to changes in total mitochondrial content, since the expression levels of COX-IV and UCP2 were not modified at any time, supporting the idea of a metabolic switch for substrates oxidation.

We observed a ∆ψ decreased at all times of diabetes, indicative of mitochondrial uncoupling. The variations in ∆ψ could be a sign of mitochondrial potential heterogeneity, as has been reported in cultured cells in high glucose conditions [27,28]. In fact, we would expect heterogeneity, since our mitochondria preparations are obtained from the different retinal cell types. Still, the decrease in ∆ψ we observed represents significant modifications in mitochondrial retinal activity as a whole. Nevertheless, the mitochondrial ATP synthesis was not modified by the diabetic condition, meaning that at these early stages, the energetic of retinal cells were not compromised.

The ∆ψ increased in response to GDP in both the normal and the 45 day-diabetes, signifying that mitochondria from normal retina have an active UCP2, which was recovered at the 45 day-diabetic stage. Even though we did not find changes in ROS production between mitochondria from normal and diabetic rat retinas, a tendency to regulate this production in the control and the 45 day-diabetic samples was observed. In agreement with these results, UCP2 activity has been involved in the “mild uncoupling” preventing superoxide production [32]. Then, the increase of COX and UCP2 activities result in retinal mitochondria adaptations leading to the decrease production of ROS.

Nonetheless, although UCP2 expression was not change, GDP did not modify ∆ψ at 7 and 20 days of diabetes, implying that UCP2 is not active and that other mechanisms participate in ∆ψ modulation, perhaps the permeability transition pore and/or adenosine nucleotide translocator (ANT). In this respect, similar results were found in the mitochondria from heart and brain long-term hyperglycemic rats, which showed a ∆ψ-enhanced susceptibility to the ANT modulation, even in the absence of changes in UCP2 expression [32–34].

In spite of the uncoupling UCP2 activity, and the growing evidence of its role regulating the flux of metabolites into the tricarboxylic acid cycle, its precise function in normal cellular physiology is still unclear [35]. Our results might indicate that UCP2 specific activity would vary depending of the physiological situation. The broad effects on coupling efficiency, ROS production and metabolic switch of UCP2 imply its complicated regulation activity in the different tissues.

Retina is constantly exposed to changes in blood glucose supply; thus, changes in ∆ψ and/or the presence of a metabolic switch, may allow a compensatory response to maintain mitochondrial physiology and thus preserve retinal function. Our results suggest that hyperglycemia decreases both glucose oxidation and the mitochondrial ∆ψ thus leading to maintain metabolic energetic requirements while avoiding oxidative stress. Although mitochondrial activity has been proposed to induce oxidative stress in diabetic rat retina [3,5,6], it has been reported that oxidative stress occurs after 45 days of diabetes in rats [7]. The same authors demonstrated that initial activation of NADPH oxidase (NOX) leads to ROS production that in turn lead to mitochondrial alterations [36]. Hence, the mitochondrial alterations observed at long-standing diabetes [5,37], most likely correspond to disease-related damage [38]. Our results open new perspectives for understanding the UCP2-modulating mechanisms of mitochondrial activity in normal retina, as well as in retinal disease.

## Supporting Information

S1 Fig Mitochondrial ultrastructure. Low magnification of mitochondrial fraction from normal retina. (A, B) Mitochondria from normal and 20 days diabetic rats.
(TIF)

S2 Fig Oxygen consumption by retina mitochondria. A representative trace of oxygen consumption, 50μg mitochondria protein (M) were incubated at 30°C, with inorganic phosphate, (Pi) 6mM; KCl, 2mM; MgCl2, 1mM; glutamate/malate, 1mM (state IV). State III (active) was induced by the addition of ADP, (100μM). KCN was added to probe the specificity of oxygen consumption by cytochrome oxidase.
(TIF)
