# Supplementary figures and images for: In the Early Stages of Diabetes, Rat Retinal Mitochondria Undergo Mild Uncoupling due to UCP2 Activity
Source: PLoS One. 2015 May 7;10(5):e0122727. doi: 10.1371/journal.pone.0122727 (PMC4423783; doi:10.1371/journal.pone.0122727)

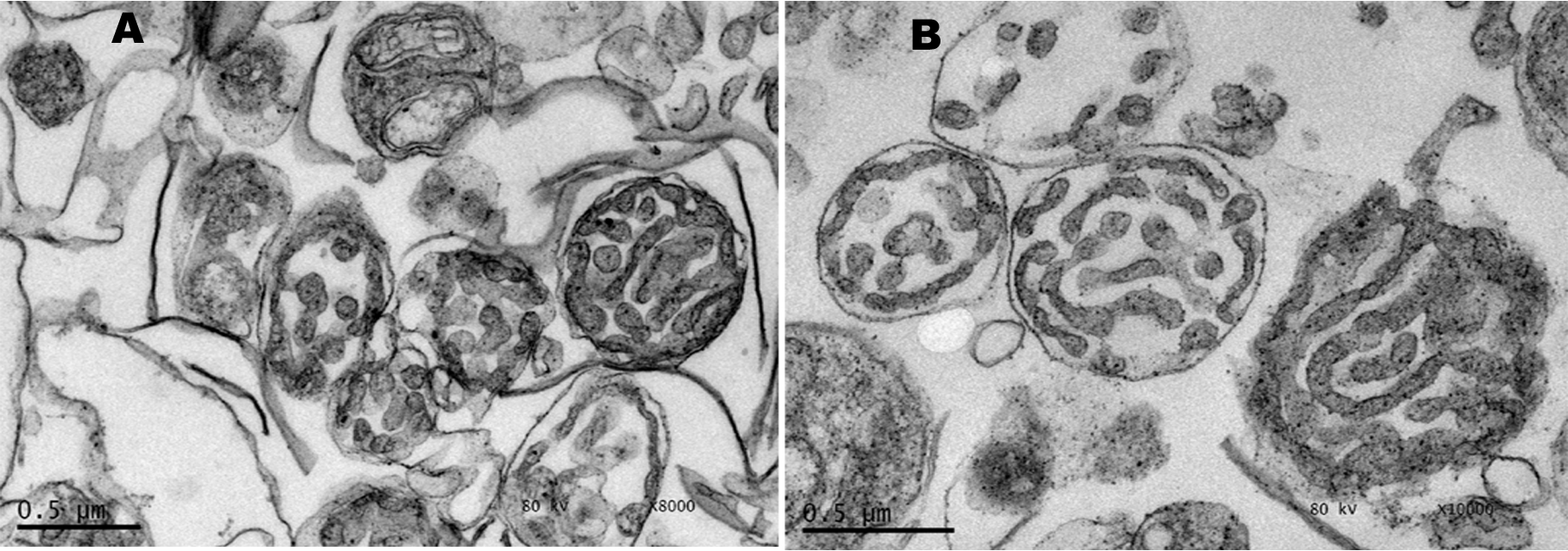

Supplement: S1 Fig — Low magnification of mitochondrial fraction from normal retina. (A, B) Mitochondria from normal and 20 days diabetic rats. (TIF) [file pone.0122727.s001.tif]

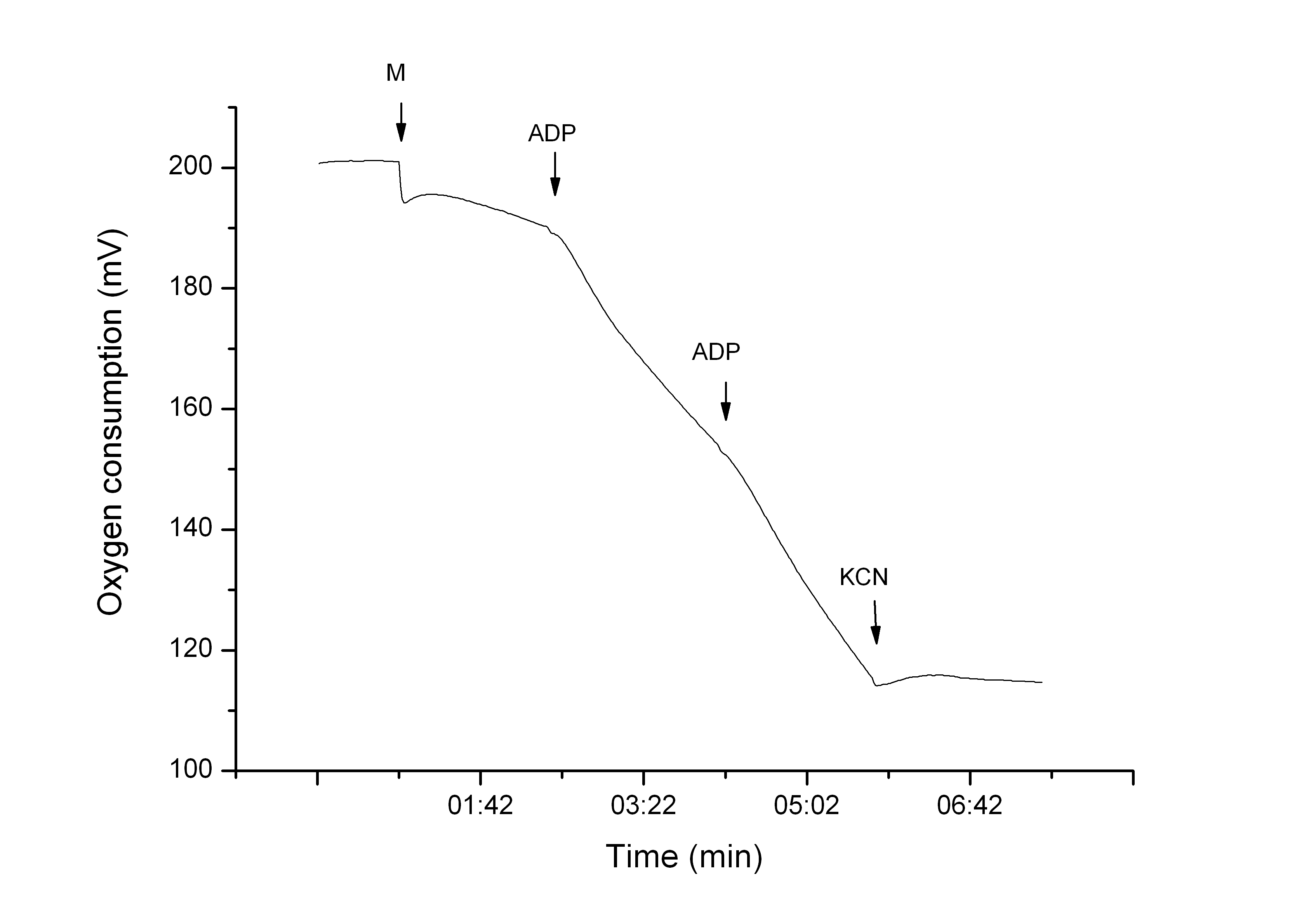

Supplement: S2 Fig — A representative trace of oxygen consumption, 50μg mitochondria protein (M) were incubated at 30°C, with inorganic phosphate, (Pi) 6mM; KCl, 2mM; MgCl2, 1mM; glutamate/malate, 1mM (state IV). State III (active) was induced by the addition of ADP, (100μM). KCN was added to probe the specificity of oxygen consumption by cytochrome oxidase. (TIF) [file pone.0122727.s002.tif]
